# Free of salt high-pressure deliming of animal hides

**DOI:** 10.1007/s11356-020-09765-2

**Published:** 2020-06-27

**Authors:** Michael Prokein, Adrian Chrobot, Manfred Renner, Eckhard Weidner

**Affiliations:** 1 Material Systems and High-pressure Technology, Fraunhofer Institute UMSICHT, Osterfelder Str. 3, 46047 Oberhausen, Germany; 2 Chair of Process Technology, Ruhr University, Universitätsstr. 150, 44780 Bochum, Germany

**Keywords:** High-pressure, CO_2_, Leather, Deliming, Ammonium nitrogen emissions

## Abstract

The wastewater pollution of tanneries is of high concern. The investigation of technologies to minimize the consumption of chemicals in the leather production process can reduce the environmental burden. We focus on the reduction of ammonium salts in the leather production process. Salt-free deliming of animal hides with compressed carbon dioxide as deliming agent is performed for the first time in a technical scale 20-L drum. As a result, CO_2_-deliming at 30 bar and 30 °C is two times faster than conventional deliming. In addition, the deliming efficiency is slightly improved. The initial calcium (Ca) content of the hides of 8 g/kg reaches the lowest value of 2 g/kg after a process time of 3 h. However, a process time of 60 min is sufficient to reach an elimination of 50 wt% of the initial lime. The resulting Ca-content of 4 g/kg after 60 min CO_2_-deliming at 30 bar is comparable with the Ca-content of conventional delimed hide. We clarify that the ampholytic character of the collagen itself enables a buffering of the pH-value at pH-7. The stable pH-value supports the selection of specific bating enzymes that decompose non-collagen proteins. No buffering salts contaminate the wastewater. The high-pressure CO_2_-deliming process has high potential to reduce wastewater emissions, save costs for chemicals, and process time in industrial beamhouse applications.

## Introduction

The production from raw animal hide to leather goods consists of 4 main categories: beamhouse, tanning, wet end, finishing. Each category includes a variety of working steps consuming high amounts of water and process chemicals (Joseph and Nithya 2009).

In the beamhouse, raw hide is prepared for the tanning process by soaking, liming, unhairing, deliming, bating, and pickling. During the preparation, hairs are removed and the hide is modified in chemical containing aqueous solutions, called floats. All chemicals used in the beamhouse are lost with the wastewater (Buljan et al. 2000, Black et al. 2013). As a result, beamhouse processing causes environmental challenges in developing countries and/or enormous costs in modern tanneries. According to estimations, the costs of environmental protection measures in European tanneries account for 4.3% of their turnover while their profit margin is just about 5% (Cotance 2012).

The use of dense carbon dioxide promises a high potential to reduce the environmental burden caused by the leather production process as already reviewed and highlighted by several authors (Sathish et al. 2016; Hu and Deng 2016; Renner et al. 2009; Renner et al. 2012). Recently, we demonstrated a CO_2_-intensified low-emission tanning process to produce high-quality leather in a 1700-L drum at 30 bar (Prokein et al. 2017). Supplementary to our previous work, we investigate the application of process features of the CO_2_-tanning process to the deliming process. The aim is a low-emission, accelerated lime removal from the hide by substituting conventional ammonium salts with dense carbon dioxide as deliming agent to prepare the hides for the CO_2_-intensified tanning process.

### Collagen

Animal hide consists of three merging layers: epidermis, dermis, subcutis. For the leather production, only collagen of the dermis is used. The other parts of the hide are utilized in other industries or have to be disposed of (Onem et al. 2018).

Collagen is with between 40 and 70 wt% of dry matter, the predominant proportion in the finished leather. As a protein, collagen is reactive towards tannins, dyestuffs, and other leather chemicals because of numerous peptide groups and its content of functional carboxy and amino groups (Reich and Taeger 2009).

Collagen is a polyampholyte with basic and acidic amino acids that can have negative and positive charges depending on the pH-value. The different charges influence the hide properties as reactivity against leather chemicals, water absorption capacity, and the orientation of the collagen fibrils. The water absorption capacity and the orientation affect the tenseness and mass transport through the cross-section of the hide, dramatically (Paul and Bailey 2003). For example, strong acid swelling at pH-3 leads to a very slow mass transport and a high degree of tenseness that irreversibly damages the collagen fibers. The high tenseness results in a decrease in strength and low leather qualities (Faber and Herfeld 1990). Therefore, proper control of the pH-value is essential in leather manufacturing to produce high-quality leather (Paul and Bailey 2003; Moog 2016).

The relation between the pH-value, the water absorption capacity, and tenseness is discussed in the “CO_2_-deliming in a 20-L autoclave at 30 bar” section in detail.

### Conventional deliming

Before the deliming process starts, hide is chemically unhaired at pH-12.5 in a sulfide- and lime-containing float. After unhairing, the hide contains calcium, which weakly bonds to the collagen, and sulfides that have to be removed (Lofrano et al. 2013).

At the high pH-value of 12.5, the unhaired hide is anionic and swollen and shows a low compressibility. The swollen fiber structure impedes chemicals and water to penetrate through the collagen structure. In this condition, excising the lime from the hide is not possible (Heidemann 1993).

The removal of the calcium is achieved by decreasing the pH-value with acidic chemicals. By acidifying the collagen at pH-12.5, the amino groups are protonated. Thereby, positive charges neutralize the negative charged collagen and the fiber structure opens facilitating chemicals to penetrate. In conventional deliming, ammonium salts are preferred for this application because they enable comparable short process times and buffer the pH at around 9. When the pH-value is not stable, the selection of convenient bating enzymes for the following working step is not possible (Covington 2009).

From an environmental point of view, ammonium salts have many disadvantages. The salts cause the formation of ammonium nitrogen (NH_3_-N) that is one of the key pollutants in the leather production process. About 6 kg of NH_3_-N originates when 1 ton raw hide is processed to leather with conventional deliming. Approximately 80% of the total NH_3_-N emission results from conventional ammonium salt deliming (Buljan 2000; Wang et al. 2012). NH_3_-N can form ammonia gas (NH_3_) causing serious diseases in contact with human beings. NH_3_-N exists also in aqueous solutions and leads to various environmental pollutions summarized by Deng et al. (2015).

### CO_2_-deliming

CO_2_-deliming is an alternative to conventional ammonium salt deliming. The mechanism of CO_2_-deliming is simple. By dissolving carbon dioxide in water, it partly converts to carbonic acid. The carbonic acid species acidify the float and remove calcium (Meregallo and Becalli 1990; Guttenberg 1994).

In contrast to the simple theoretical mechanism, the use of CO_2_ for deliming at ambient conditions has several challenges. First, the solubility of carbon dioxide in water is low leading to unacceptable operating times compared with conventional ammonium deliming. The solubility increases at cold conditions. However, enzymes needed for the following bating process show their optimal activity between 30 and 40 °C. In addition, the penetration of the dissolved carbon dioxide through the cross-section of the hide is slow which impedes treating thick hides. In addition, controlling the amount of available gas to keep the pH-value stable without using buffering salts is not applicable (Deng et al. 2015; Covington 2009; Flowers 2002).

Due to the challenges, CO_2_-deliming is industrially only performed in combination with ammonium salts. Thereby, about 75% of NH_3_-N emission caused by conventional deliming can be avoided; however, 25% remain (Black et al. 2013).

## Research issue

**Fig. 1:**
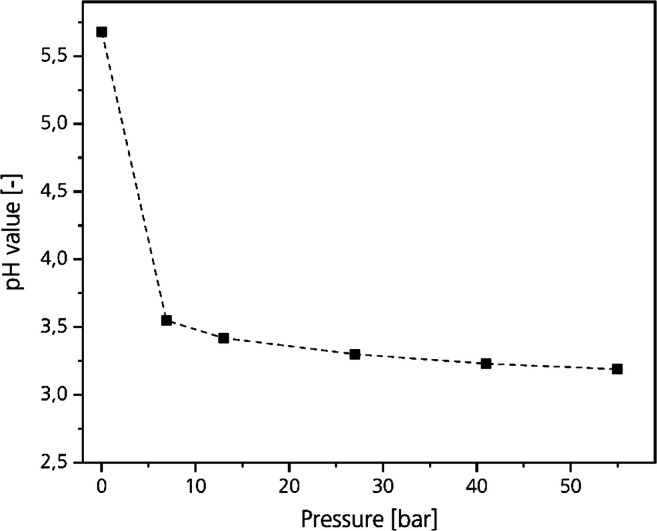
pH-value of water saturated by CO_2_ depending on the CO_2_ partial pressure at 32 °C (Meyssami et al. 1992)

The most important challenge preventing salt-free CO_2_-deliming is the low solubility of CO_2_ in water at ambient conditions (“CO_2_-deliming” section). An opportunity to overcome this challenge at applicable temperatures (30 to 40 °C) is to increase the pressure. For example, the solubility of CO_2_ in water at a temperature of 30 °C and a pressure of 50 bar is with 47.5 g/l approximately 40 times higher compared with a CO_2_-partial pressure of 1 bar (Duan and Sun 2003). Due to the pressure-induced solubility and the carbonic acid formation, the pH-value of water decreases to about 3 above a pressure of 30 bar (Peng et al. 2013; Mohamed et al. 2011). Figure 1 shows the pH-curve of CO_2_-saturated water at 32 °C according to Meyssami et al. (1992).

A reduction of the collagens’ pH-value to pH-3 at 30 bar would cause acid swelling and irreversibly damage the hide structure (“Collagen” section). However, the solubility of CO_2_ in water and thereby the effect on the pH-value can be influenced by various chemicals or materials (Chuang and Johannsen 2009). Since collagen is a polyampholyte with an enormous binding capacity for acids, we assume that the pH-value in a CO_2_ pressurized system shows a different trend in a collagen-containing float compared with pure water. Since the design of conventional tanning equipment is only convenient for ambient conditions, high-pressure deliming and the effect of pressurized CO_2_ on the pH of water containing unhaired hide at pH-12.5 has not been investigated, yet.

Beyond deliming, several authors considered the application of dense carbon dioxide in various working steps of the leather production process. However, nearly all of the work focused on lab scale experiments with supercritical CO_2_ linked to pressures above 74 bar (Hu and Deng 2016; Sathish et al. 2016). To build equipment that is able to operate with supercritical CO_2_ for industrial beamhouse applications is challenging. The main problem is the needed diameter for the high-pressure equipment to be able to process up to 12 t of hide in a volume of more than 20 m^3^ per batch comparable with conventional beamhouse operations (Renner 2015).

The CO_2_-intensified tanning process developed by Renner et al. is the only workstep in the leather production chain that was demonstrated in pilot scale (500 kg full bovine hide tanning). The reason for this is the moderate operating pressure of 30 bar, which enables the development of high-pressure rotating drums for industrial applications. The process combines the positive characteristics of the conventional chromium tanning process like high performance and quality with the possibility of saving process time, chromium tanning agent, and water, especially wastewater. The process is commercially attractive as the return on investment is less than 2 years (Weidner 2018) (Prokein et al. 2017) (Renner et al. 2012) (Renner et al. 2009).

Derived from the state of research, the aim of this work is to adapt features of the CO_2_-intensified tanning process to the deliming process. The challenges of the conventional CO_2_-deliming process caused by low solubility of CO_2_ at ambient conditions should be overcome by deliming at 30 bar. The influences of the evaluated pressure on the pH-value including linked changes of collagen properties and the lime removal efficiency are considered.

## Materials and methods

### High-pressure equipment

#### High-pressure view cell

**Fig. 2:**
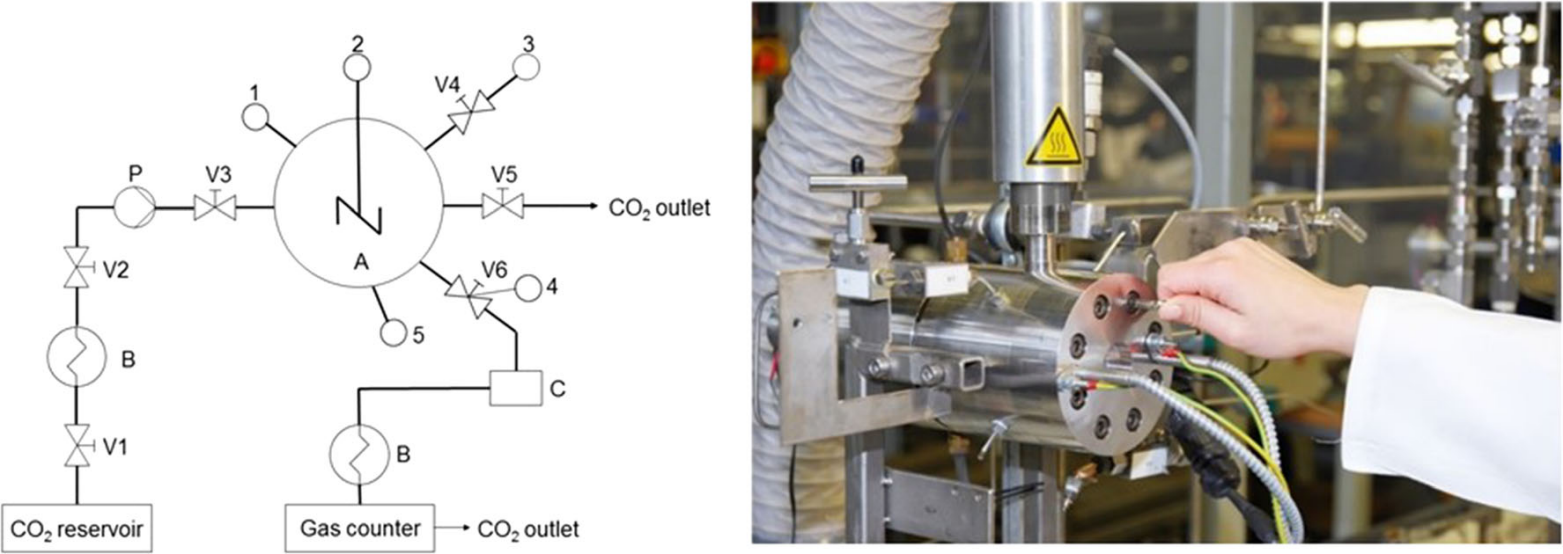
Schematic diagram and a photograph of the 63-ml high-pressure view cell; A, cell; B, heat exchanger; C, separator; P, pump; V, valve; 1, pressure sensor; 2, stirrer; 3, inlet for liquids; 4, temperature sensor; 5, temperature control

The influence of pressurized CO_2_ on the pH-value of pure water and floats containing collagen were carried out in a high-pressure view cell. Figure 2 shows a schematic diagram and a photograph of the equipment. The cell has a volume of 63 mL, a maximum pressure of 300 bar, and a maximum operation temperature of 260 °C.

To pressurize the cell, a high-pressure pump compresses CO_2_ that is stored at 60 bar in a CO_2_ tank. By opening valve 3, the cell can be manually pressurized to the desired pressure. Valve 4 enables the depressurization.

A sapphire window positioned at the front of the view cell enables the observation of the process. A lamp installed behind a second rear window lights up the interior of the cell. A stirrer can optionally improve the mass transport.

#### Twenty liter high-pressure drum

**Fig. 3:**
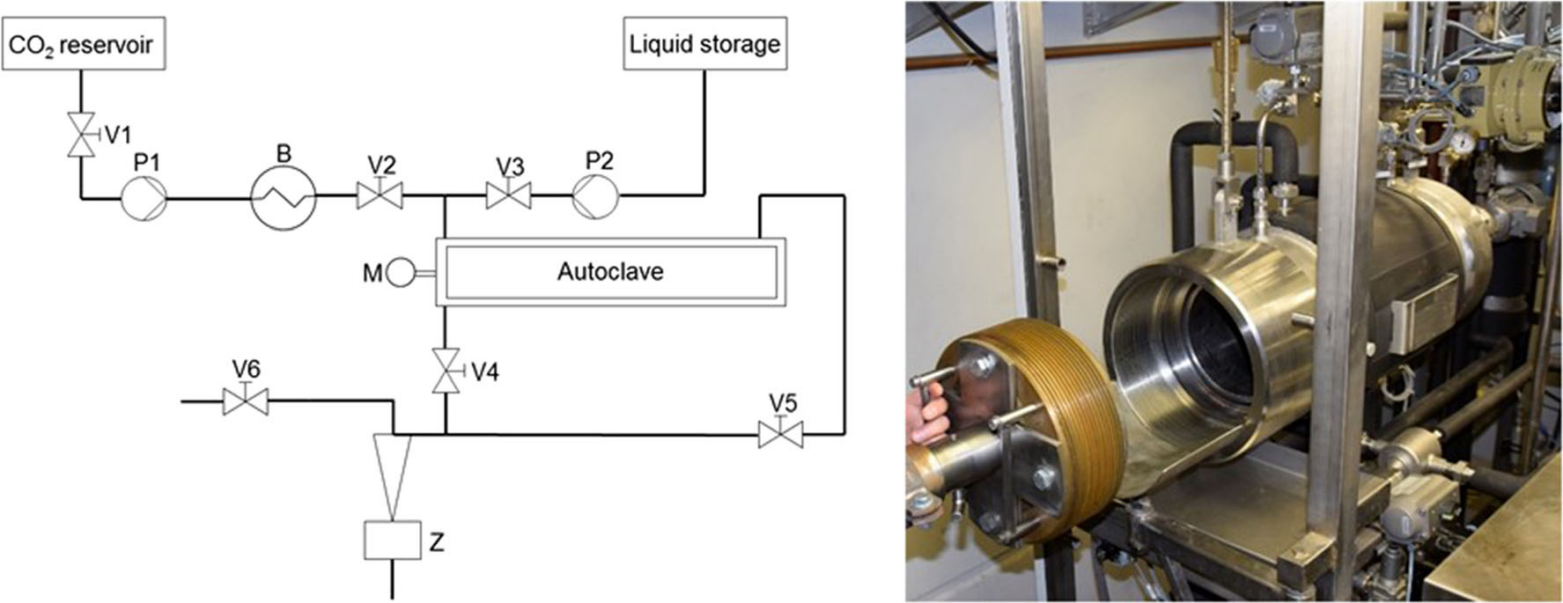
Schematic diagram and photo of the 20-L autoclave; B, heat exchanger; P, pump, M, motor; Z, cyclone; V, valve

Deliming test series were carried out in PLC-controlled 20-L high-pressure equipment. Figure 3 shows a schematic diagram and a photograph of the apparatus. A rotatable basket is installed inside a horizontal, cylindrical vessel. In the rotating basket, hides are processed comparable with industrial procedures. Pikes inside the basket ensure that the hides move around continuously during deliming.

CO_2_ is stored in a CO_2_ tank at 60 bar. A high-pressure pump (P1) allows pressurization of the reactor with pressures between 30 and 280 bar. A second pump (P2) allows the pumping of liquids against the maximum pressure of the reactor. Liquids are stored in a separate reservoir. At the end of the process, the reactor is depressurized and the CO_2_ is separated from the liquids.

### Hide

Bull hide with a surface area of approximately 7 m^2^ and a wet weight of about 25 kg was used. Conventional unhaired hides at pH-12.5 and conventional delimed hides at pH-8.5 were provided by »Lederfabrik Josef Heinen GmbH & Co. KG«. The split was 4.5 mm. The unhaired hides were used for the high-pressure deliming test series. The conventional delimed hides were used as reference.

As already described in previous work, a bovine hide is divided into several parts with different characteristics and fiber structure (Prokein et al. 2017). The parts can be defined in terms of butt, belly, and neck. The part with the highest quality and the most regular structure is the butt, called croupon.

For each experiment in the 20-L equipment, 10 hide samples with a size of 15 cm × 20 cm each, were cut out of the croupon. The average weight of the samples was 199.4 g with a standard deviation of 14.7. For the trials in the view cell, smaller cylindrical samples of some grams were punched out of the croupon.

### Analytics

Hide is a material characterized by natural deviations. To minimize the influence of the deviations, all 10 hide samples of each 20-L trial were considered for the analytics apart from the residual calcium and sulfide determinations described in the “Lime- and sulfide-contents of hide and float” section. In addition, we performed double determinations for each experiment. From all results, average values including standard deviations were determined. As reference, the conventional delimed hides were equally analyzed to assess the tenseness and compressibility (“Tenseness and compressibility” section) and lime- and sulfide-contents (“Lime- and sulfide-contents of hide and float” section).

#### Hide weight and thickness

The weight and the thickness of the hides before and after CO_2_-deliming indicate whether the charges of the collagen and thereby the fiber structure and water absorption capacity changed. Therefore, the weights and the thicknesses of all samples were measured before and after the high-pressure CO_2_-deliming process.

The thickness of each sample was determined by considering 4 marked areas per sample before and after deliming to calculate an average value including standard deviation.

#### Tenseness and compressibility

The change in tenseness and compressibility of the hides due to CO_2_-deliming is determined by a principle of Herfeld and Schubert. A cylindrical specimen is placed on a defined area of a hide sample. The ratio between the specimens weight and surface area is 220 g per cm^2^. The force of the specimen reduces the thickness of the hide depending on the circulation period.

A meaningful parameter to characterize the tenseness of the hide is defined as the total percentage compressibility value *E* according to formula 1.

1 $$ E=\frac{IT-T4}{IT}\times 100 $$

The value *E* represents the percentage decrease in thickness after 4 min of pressing time, relative to the initial thickness. In the formula, *IT* stands for the initial thickness and *T*4 for the thickness after 4 min of pressing. The smaller the value *E*, the smaller is the compressibility indicating the ratio between positive and negative charges inside the collagen (Zissel and Herfeld 1989).

#### The pH-values of hide and floats

The pH-value of the floats from the 20-L trials was directly measured after depressurization and separation with a pH-probe »type 911« from »Knick Elektronische Messgeräte GmbH und Co. KG«.

To assess the pH-value of the hides, 3 DIN A5 samples were sammed and the pH was measured with the pH-probe. An average value including standard deviation was determined.

For the determination of the pH-value in the cross-section, 3 hide samples per trial were cut and wetted with different liquid pH-indicators ordered at Sigma-Aldrich: phenolphthalein (colorless between pH-0 and 8.2), bromothymol blue (change of color from 7 to 8), bromophenol blue (change of color from 3 to 4.6).

The liquid pH-indicators bromothymol blue and bromophenol blue enabled the assessment of the pH-value of aqueous solutions in a pressurized view cell.

#### Lime- and sulfide-contents of hide and float

To assess the lime and sulfide content, the calcium and sulfur concentrations were measured by inductively coupled plasma optical emission spectrometry (ICP-OES). The difference in the molecular weight between sulfur and sulfide was neglected. The initial Ca- and S-contents of the unhaired hides, the conventional delimed hides, and the high- pressure CO_2_-delimed hides were considered. In addition, the enriched Ca- and S-contents in the process water were also measured by ICP-OES. For each trial, a double determination was performed.

### High-pressure procedure

#### Optical assessment of the pH-value

Distilled water containing bromophenol blue or bromomethyl blue was filled in the view cell. Different amounts of collagen at pH-12.5 were added to investigate the influence of the ratio between water and collagen. Afterwards the cell was pressurized with 5 bar/min. When the color of the indicator changed during the pressurization, a photo was taken and the pH-value assessed. One test series was performed without adding hide to compare the results of CO_2_-saturated water with available literature data to assess the viability of the procedure. To check whether the collagen or the chemicals inside the collagen influence the color of the pH-indicators by chemical reactions, trials without CO_2_ pressure were performed.

#### High-pressure CO_2_-deliming

The high-pressure deliming process was performed in a 20-L drum. The DIN A5 hide samples were placed in the rotating basket. The drum was closed and 200 wt% of water calculated on the hide weight was pumped in. Two hundred weight percent of water is usually used in deliming. Afterwards the reactor was pressurized to the desired pressure of 30 bar. The pressure was kept constant for the defined process time. The considered process times were 25, 60, 120, 180, and 360 min. The temperature was 30 °C comparable with conventional deliming and bating. In the end of the process, the drum was depressurized with 1.5 bar/min. At this depressurization kinetics, the hide structure is not damaged, known from previous work. The rotation of the basket was 5 rpm to achieve mechanical movement of the hide. Faster rotations were avoided to protect the surface of the hide samples. After the process, the remaining floats and the treated hide samples were assessed as described in the “Analytics” section. Each experiment was performed two times.

## Results and discussion

### Optical pH-assessment of the float containing hide

#### Influence of the ratio between water and collagen

The pH-value of pure water decreases to about 3 by increasing the CO_2_ pressure to 30 bar (Fig. 1). If the pH-value of collagen pressurized with CO_2_ would also decrease to 3, acid swelling would irreversibly damage the hide fibers. However, collagen is able to bind protons to the functional amino and carboxy groups (“Collagen” section). The pH-values of aqueous solutions containing collagen pressurized by CO_2_ are not known. We assessed which pH-values result in a CO_2_-saturated float containing collagen at pH-12.5 up to 100 bar under variation of the float length. The float length is the ratio between hide and water in weight percentage.

**Fig. 4:**
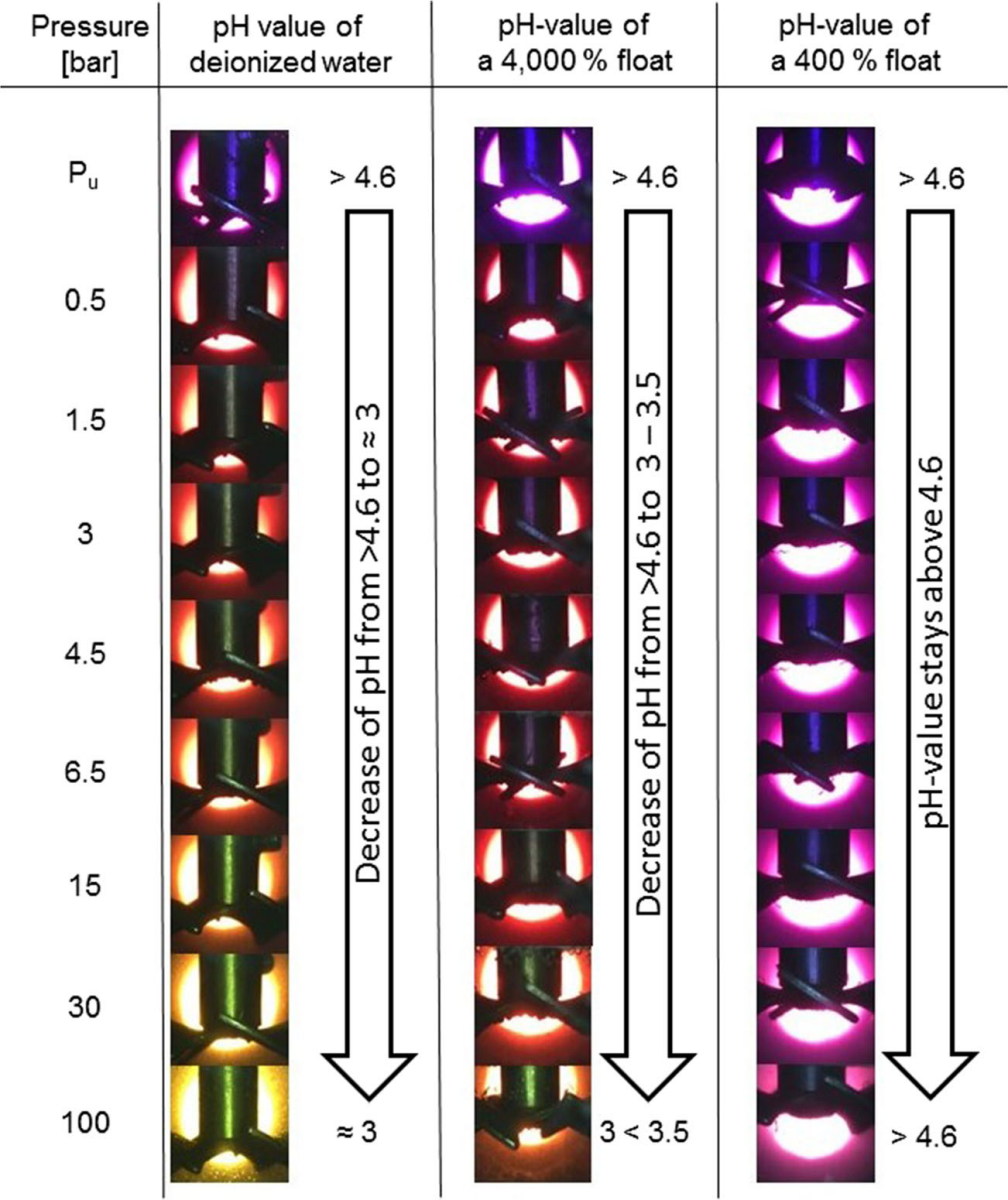
Pictures of the optical assessment of the pH-values of water and aqueous solutions containing a different amount of collagen in a high-pressure view cell at pressures between P_u_ and 100 bar

In Fig. 4, the optical pH-assessment by using bromophenol blue as indicator is illustrated in pictures. On the left side are the pictures of the test series without collagen. At ambient conditions, the color of the solution is violet (pH > 4.6). Without increasing the pressure by CO_2_, the color does not change. By a slight increase in pressure to 0.5 bar, the color changes to red (4 < pH < 4.6). By a further increase in pressure, the color of the solution changes from red to orange to yellow. At 30 bar, the color of the solution is yellow to orange indicating a pH-value slightly above 3. By increasing the pressure to 100 bar, a pH-value of about 3 is reached.

In the middle of Fig. 4 are the pictures of the test series by using a float length of 4000 wt%. A float length of 4000 wt% means 10 mg of collagen per 40 g of water. Compared with the test series performed without collagen, the pH-values are higher. The color changes from violet to red at a pressure of 0.5 bar. However, the transition from red to orange becomes visible at pressures above 15 bar. Without collagen, the transition from red to orange was already observed above 1.5 bar. At 100 bar, the pH-value is between 3 and 3.5. There is no color transition from orange to yellow.

On the right side of Fig. 4 are the results of the test series with a float length of 400 wt% or 100 mg of collagen per 40 g of water, respectively. Compared with the previous tests, the pH-values at the different pressure steps are clearly higher. At 400 wt%, the pH-value does not decrease below 4.6 independent from the pressure. At a typical float length of 200 wt%, no color transition was observed.

The optical assessment of the pH-value of pure water pressurized with CO_2_ is in agreement with data from literature (Meyssami et al. 1992; Mohamed et al. 2011; Peng et al. 2013; Bortoluzzi et al. 2011; Haghi et al. 2017). This indicates the possibility to visualize the pH-value of water with liquid pH-indicators in a pressurized system. The addition of collagen did not result in a color transition without pressurizing the float. We derive that the collagen and the chemicals inside the collagen do not influence the color of the indicator and conclude that the procedure is feasible.

It has to be noted that the optical assessment of the pH-value can be connected to different errors (Stippl 2005). The use of pH-probes designed for high-pressure applications enables measurements that are more precise. However, measurements with pH-probes in pressurized systems require very slow pressurizing kinetics to avoid a damage of the probes and are hard to handle (Mohamed et al. 2011). Since the focus of this work is the deliming of animal hides in technical scale where the pH-values of the hide samples are measured with pH-probes (“Influence of CO_2_ deliming on the pH-value of hide and float” section), we assess the accuracy of the optical assessment as sufficient to estimate how the pH-values of the floats change depending on CO_2_ pressure and ratio between water and collagen.

The optical pH-indications show that by decreasing the float length, the pH-values decrease at comparable pressures. The reason is the acid binding capacity of collagen. By decreasing the float length, the concentration of functional groups that are able to bind protons increases. Consequently, collagen buffers the pH-value of water pressurized with CO_2_. However, when the ratio between water and collagen is high, the deviation between the pH-values decreases. This is due to a high surplus of water that increases the availability of dissolved carbon dioxide forming carbonic acid to protonate the functional groups and decrease the pH-value.

No comparable publications that consider the pH-value of aqueous solutions containing collagen pressurized by CO_2_ are available. However, researchers observed similar trends in pressurized CO_2_-water systems containing other buffering salts. Andersson et al. investigated the pH-values of ammonium acetate dissolved in water pressurized by CO_2_. At a constant pressure of 80 bar, the pH-values increased from 3.5 to 4.5 by increasing the concentration of the buffering salt from 2 to 30 mM (Andersson et al. 2018). Li et al. showed that in saturated sodium hydrogen carbonate solutions, the pH-value increases at a CO_2_ pressure of about 90 bar from 4.2 to 6.6 by increasing the salt concentration from 0.01 to 1 mol/kg (Li et al. 2018).

A float length of 4000 wt% theoretically allows the reduction of the pH-value directly from 12.5 to about 3. This enormous pH-drop in one working step could be beneficial for the leather production process. The possible advantage is explained by the fact that before tanning the pH-value has to be reduced to 3 by sulfuric and formic acid (Jia et al. 2016). A substitution of these acids with carbon dioxide would reduce sulfate and formate emissions that are of high concern in the leather industry (Sundar et al. 2002; Galiana-Aleixandre et al. 2011). However, a water consumption of 4000 wt% is from many points of view not useful. For example, enormous drum volumes and amounts of salt to avoid acid swelling would be needed. Therefore, a typical water consumption of 200 wt% should be addressed to realize an industrial process.

At a float length of 400 and 200 wt%, bromophenol blue did not show a color transition independent from the applied pressure. Since a water consumption of 200 wt% and a pressure of 30 bar are useful parameters for an industrial application, we assessed the pH-values at these parameters with bromothymol blue in an additional test series.

#### Influence of pressure at a constant float length of 200 wt%

**Fig. 5:**
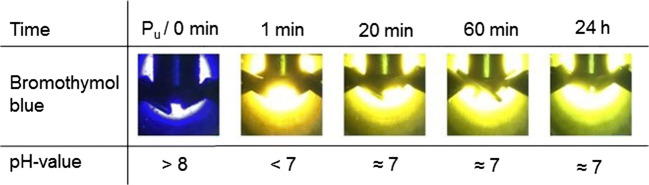
Pictures of the optical assessment of the pH-value of a collagen-containing float with a float length of 200 wt% with bromothymol blue at 1 bar

**Fig. 6:**
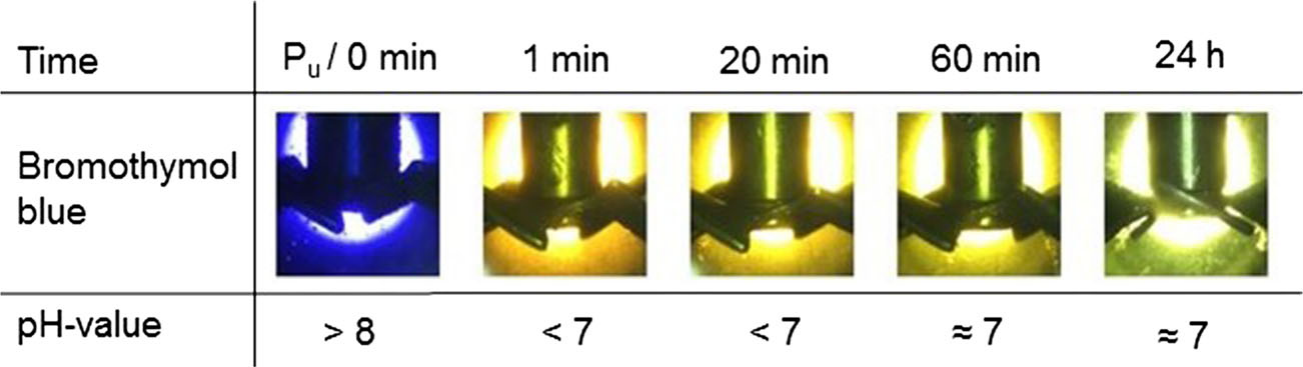
Pictures of the optical assessment of the pH-value of a collagen-containing float with a float length of 200 wt% with bromothymol blue at 5 bar

**Fig. 7:**
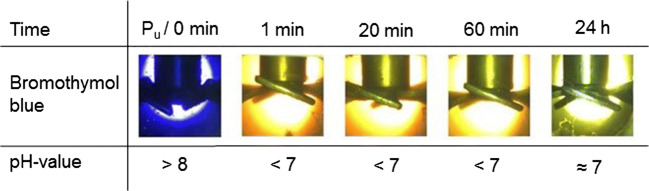
Pictures of the optical assessment of the pH-value of a collagen-containing float with a float length of 200 wt% with bromothymol blue at 30 bar

The results of the test series with a float length of 200 wt% were performed with pressures of 1, 5, and 30 bar. Figures 5, 6, and 7 show the photos of the optical assessment of the time depending pH-values of the collagen containing floats with bromothymol blue.

In all test series, the initial color without applying pressure is violet indicating a pH > 8. This was expected because the initial pH of the collagen was 12.5. Without raising the pressure by CO_2_, the color of bromothymol blue did not change. By increasing the CO_2_ pressure, the liquid indicator directly changed the color from violet to yellow at all considered pressures. A yellow color of bromothymol blue indicates that the pH-value decreased below 7. Afterwards the color changed during all experiments to green. The greenish color indicated a pH-value of about 7. The difference between various pressures was that the transition from yellow to green was faster at lower pressures. At 1 bar, the transition started after 20 min. By increasing the pressure to 5 bar, the transition started after 60 min. At 30 bar, it took 7 h until the transition started. Considering the results after 24 h, the pH-values at all tested pressures were similar independent from the applied pressure.

In the previous test series, we showed that the amount of water and at the same time the availability of dissolved CO_2_ leads to a decrease of the pH-value. An increased pressure should also increase the solubility and thereby the availability of CO_2_ molecules. A pressure-induced increase of available CO_2_ molecules should decrease the pH-value (Bahadori et al. 2009). However, after reaching equilibrium, the color difference and at the same time the difference of the pH-values between the test series is low.

We suggest that the pH-value does not decrease significantly by raising the pressure from 1 to 30 bar because of the buffering effect of the collagen. Collagen has an enormous binding capacity for acids (Brown 2013). For example to saturate the basic groups of 10 tons conventional unhaired hide, 120 kg of pure sulfuric acid is needed (Covington 2009). Since sulfuric acid is a strong diprotic acid, it derives that about 2.5 thousand mole of oxonium ions are needed to saturate the basic groups of 10 tons unhaired hide. Consequently, in the conventional leather production process, a change of the pH-value and the collagens’ reactivity requires high amounts of chemicals (Buljan et al. 2000). At a CO_2_ pressure of 30 bar and at 30 °C, the pH-value of water decreases to 3. At pH-3, the oxonium ion concentration is about 0.001 mole per liter. At a float length of 200 wt%, 20 mole of oxonium ions was available to protonate 10 tons of collagen in 20,000 l of water; however, 2.5 thousand is needed. Therefore, it is not surprising that the pH-value of a pressurized aqueous solution differs from an aqueous solution containing collagen as already discussed in the previous section.

It has to be noted that the solubility of CO_2_ and the dissociation rate are strongly influenced by collagen. Otherwise, the influence on the pH-value would be negligible. The carbon dioxide dissolves in the aqueous solution and dissociates depending on the pH-value partially to H^+^ and HCO_3_^−^ or 2H^+^ and CO_3_^2−^ (Sontheimer et al. 1980). The H^+^ protonate the reactive groups of the collagen. Thereby, more carbon dioxide dissociates in a solution containing collagen compared with pure water. However, in this complex collagen-water system, we observe that the pH-value is stable at around 7 and does not decrease to 3 as it would happen in water without collagen.

Concurrently to the pressure, the float length influences the pH-value (Fig. 4). However, the float length is a parameter that is hard to change in an industrial deliming process. The surface of swollen unhaired hide at pH-12.5 is sensitive against mechanical influences. A minimum of water is needed to protect the hide. In addition, water is needed to wash out the lime, sulfide, and other dissolved components (Zissel and Herfeld 1989; Covington 2009). A longer float can lead to a further decrease of the pH-value and enable the saving of conventional acids. However, as already discussed in the previous section, a longer float is connected with higher loads and with higher water consumption.

The tests show that at industrial-applicable parameters of 30 bar and a float length of 200 wt%, a stable pH-value of 7 results. We assume that after 24 h the pH-value does not change anymore. This assumption will be stressed in the “The pH-values of hide and floats” section. A stable pH-value is essential to choose bating enzymes that decompose non-collagen proteins in the following working step (“Conventional deliming” section). At pH-7, possible enzymes that show a high activity are for example papain or casein (Zissel and Herfeld 1989).

To reach a constant pH-value took several hours in the view cell. For an industrial application, the duration for deliming should not exceed 2 h (Deng et al. 2015). However, the process conditions in the view cell differ a lot from the conditions in a rotating drum in which the mechanical action can improve the mass transport. Therefore, trials in a 20-L autoclave with a rotating basket were performed in technical scale.

### CO_2_-deliming in a 20-L autoclave at 30 bar

The technical scale experiments were performed as described in the “High-pressure CO_2_-deliming” section. The experiments enable to analyze hide processed at 30 bar in a rotating basket comparable with a conventional rotating drum. The hide samples and the process floats were analyzed as described in the “Analytics” section.

#### Influence of CO_2_-deliming on the hide weight and thickness

Before CO_2_ deliming is performed, the unhaired hide at pH-12.5 is strongly swollen and has a high water content. Depending on the pH-value, the water content and thereby the weight of the hide changes (“Collagen” section).

**Fig. 8:**
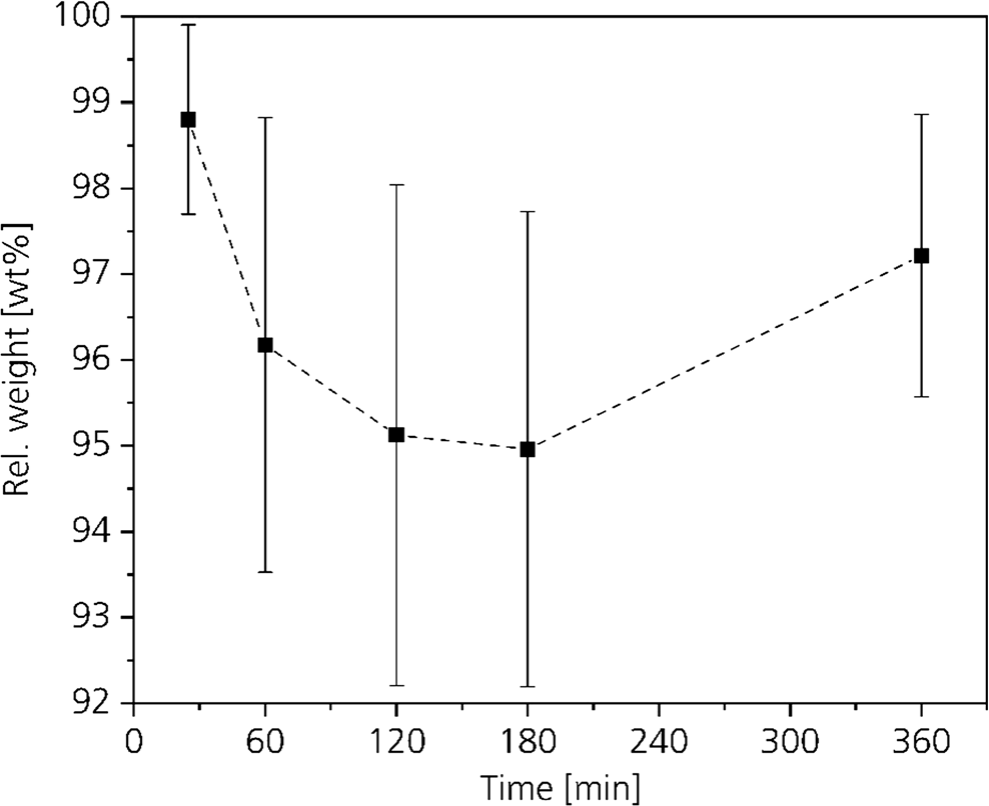
Influence of CO_2_-deliming time on the weight loss

Figure 8 shows the weight loss in percentage based on the initial weight of the unhaired hide samples depending on the process time after CO_2_-deliming. The weight loss on average is between 2 and 5 wt%. After the shortest process duration of 25 min, the lowest weight loss of 1.2 wt% with a standard deviation of 1.1 resulted. By increasing the process time to 180 min, the weight loss increases and reaches a maximum of 5 wt% with a standard deviation of 2.8. At a process time of 360 min, the weight loss was 2.8 wt% with a standard deviation of 1.6. Except for the shortest process time, all calculated average values are within all standard deviations. Hence, we conclude that increasing the process time above 60 min has no influence on the weight loss.

**Fig. 9:**
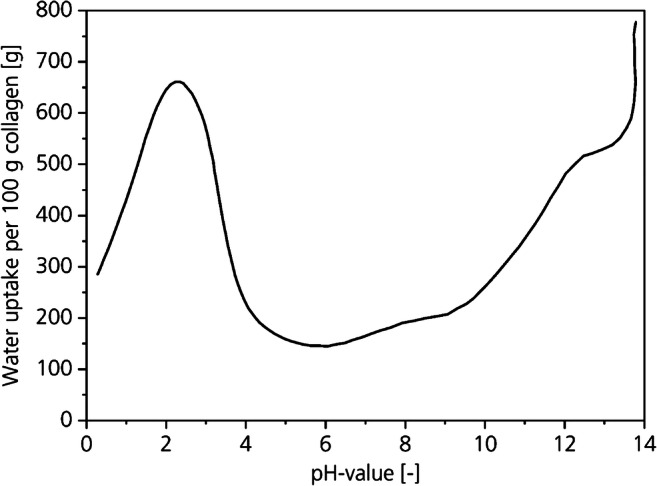
Water absorption capacity of collagen depending on the pH-value (Covington 2009)

Figure 9 shows the water absorption capacity of the collagen depending on the pH-value. The water content of collagen can increase from 60 wt% (150 g water per 100 g of dry collagen) from the isoelectric point to about 85 wt% (650 g water per 100 g of dry collagen) due to acid or basic swelling.

From the weight loss presented in Fig. 8 in relation to the water absorption capacity shown in Fig. 9, we derive that the pH-values decreased during all CO_2_-deliming trials. The charges inside the collagen are equalized in the direction of the isoelectric point.

**Fig. 10:**
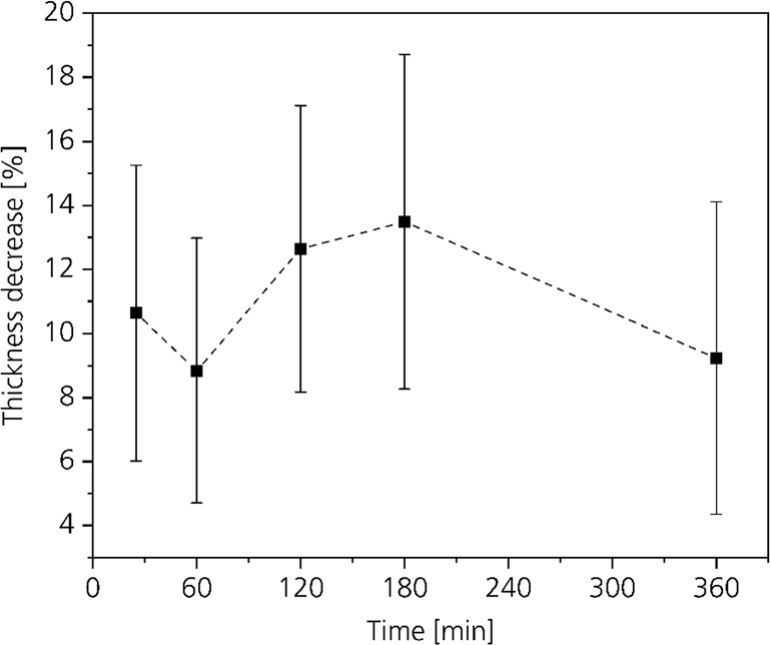
Influence of the CO_2_-deliming time on the hide thickness

Figure 10 shows the change in thickness of the samples depending on the process time. On average, the thickness of all samples decreased between 8.8 and 13.4% with standard deviations between 4.1 and 5.2. All average values are within all standard deviations. Compared with the weight loss, the decrease in thickness is in good agreement. When the tenseness is reduced and water and chemicals leave the hide, it is not surprising that also the thickness of the hide decreases.

The results of the weight loss and thickness of the shortest experiment do not perfectly correlate. However, considering the high standard deviations and the natural character of the material, we pay no further attention to this deviation from the other results.

#### Influence of CO_2_-deliming on compressibility

**Fig. 11:**
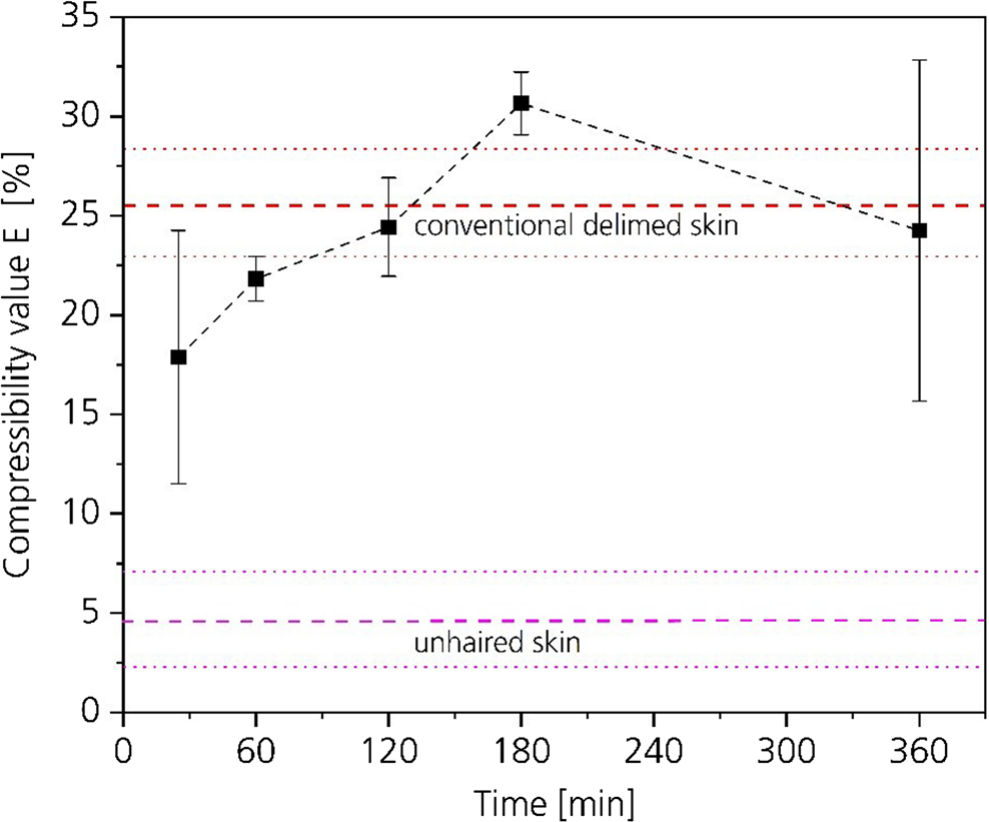
Influence of CO_2_-deliming time on the collagens’ compressibility

The compressibility value *E* indicates the tenseness of the collagen fibers. Figure 11 shows the *E*-values of all 30-bar CO_2_-deliming experiments, of the initial unhaired hide and as reference of a conventional delimed hide. The *E*-value of the unhaired hide is 4.7% with a standard deviation of 2.4. The conventional delimed hide has a compressibility value *E* of 25.5% with a standard deviation of 2.6. The values show an enormous difference between the compressibility of unhaired hide at pH-12.5 and delimed hide with a pH-value of 9 as described in the “Collagen” section.

**Fig. 12:**
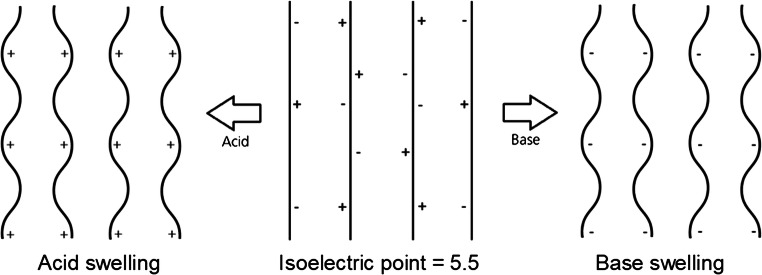
Schematic mechanism of the influence of the charges on the orientation of the collagen fibers

The influence of the pH-value on the tenseness relates to the same mechanism as for the weight and thickness. Figure 12 explains the mechanism of the influence of the charges on the orientation of the collagen fibers, schematically. The mass transport in the cross-section of the hide depends on the orientation and tenseness of the collagen fibers influenced by the charges of the reactive groups. Below or above the isoelectric point, the portion of identical charges in the hide increases. The same charges repel each other. Thereby the fibers tense up, the water absorption capacity increases, the hide swells, and its compressibility decreases. As further from the isoelectric point as slower is the mass transport through structure of the hide and as higher are the *E*-values (Moog 2016; Zissel and Herfeld 1989).

After a process time of 120 min, all compressibility values of the 30 bar CO_2_-deliming experiments are within or above the standard of the conventional delimed hide. This indicates that the orientation of the collagen fibers and the collagens’ charge of the CO_2_-delimed samples are comparable with conventional delimed hide. After a process time of 60 min, the compressibility is already close to the conventional delimed hide.

The conventional hide was processed in industrial scale in a 12-t batch. The mechanical action in industrial scale is stronger compared with the mechanical action in the 20-L equipment. Thereby, we assume that in industrial scale a process time of 60 min would be sufficient to reach an *E*-value comparable with conventional delimed hide.

#### Influence of CO_2_ deliming on the pH-value of hide and float

A stable pH-value is essential for the deliming process. The reason is that in the following bating step, enzymes lose their activity when the pH-value varies. The assessment of the pH-value of the water pressed from the cross-section of all hides resulted in an equal pH-value slightly above 7, independent from the duration of the process.

**Fig. 13:**
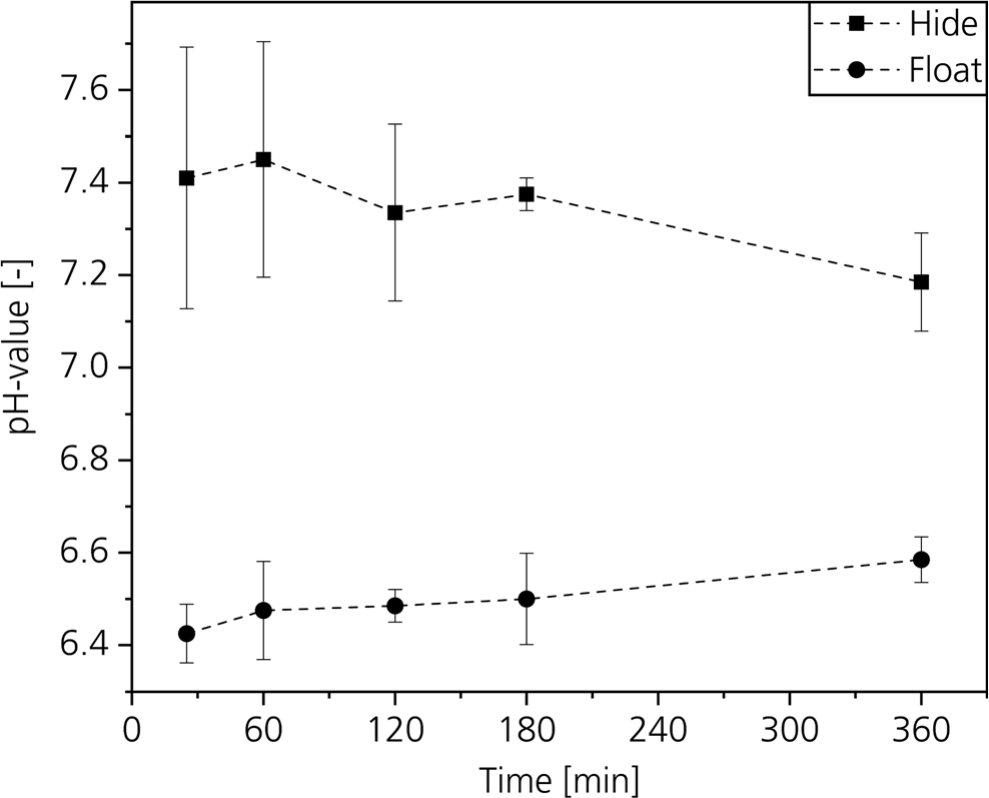
Influence of the process time on the pH-values of collagen and float

Figure 13 shows the pH-values of the hide samples and of the floats of the different time depending experiments assessed by a pH-probe. After a process time of 25 min, the pH-value of the hide was 7.4 with a standard deviation of 0.28. The float pH was 6.4 with a standard deviation of 0.06. By increasing the deliming time to 360 min, the pH-value of the hide slightly decreases to 7.2 with a standard deviation of 0.1 while the pH-value of the float increases to 6.6 with a standard deviation of 0.05. Based on the experiments in the view cell with a constant float of 200 wt% described in the “Influence of pressure at a constant float length of 200 wt%” section, we assume that the pH-values of the float and the hide meet after reaching equilibrium at a pH-value of about 7.

A decrease of the pH-value from 12.5 to 7.4 within the whole cross-section of the hide is already reached within 25 min. The difference between the pH of the float and the hide after this period is just 1. We assume that the pH-value after a time of 25 min is already stable enough to initiate bating, because enzymes that have a high activity between pH-6 and 8 are available (Zissel and Herfeld 1989). Considering that bating takes usually 30 min, a time saving of at least 50% is possible. Conventional ammonium deliming takes 2 h (Širvaitytė et al. 2006).

#### Residual lime and sulfide content

**Fig. 14:**
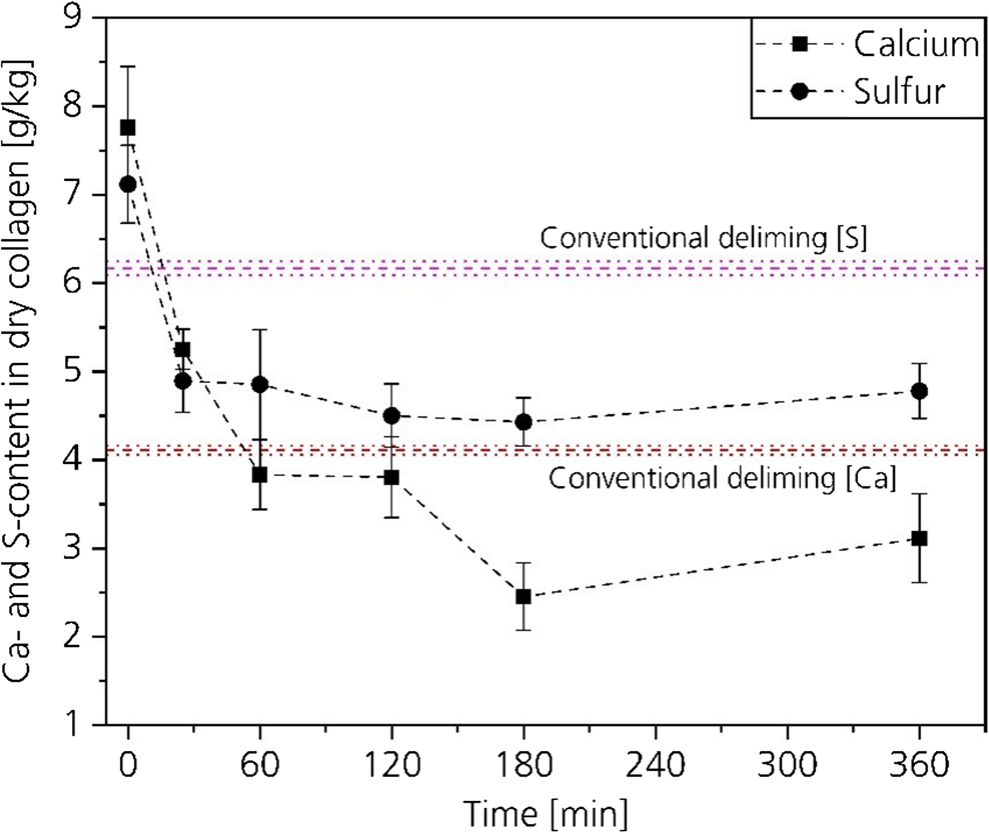
Residual Ca- and S-contents in dry collagen

**Fig. 15:**
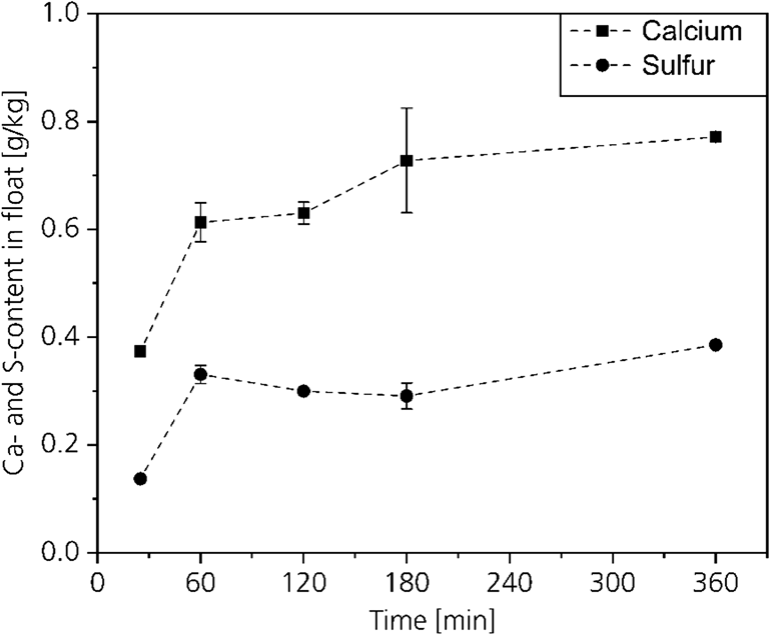
Calcium and sulfur contents in the residual float

The most important in the deliming process is that the lime is removed from the hide. Figure 14 shows the Ca- and S-contents of conventional delimed hides, unhaired hides, and of the CO_2_-delimed hides at 30 bar based on the dry weight at different process times. Figure 15 shows the Ca- and S-contents based on the weight of the residual float in grams per kilogram. As already described in the “Lime- and sulfide-contents of hide and float” section, the difference in the molecular weight between sulfur and sulfide is neglected.

The Ca- and S-contents of Figs. 14 and 15 show opposite trends. The contents in the collagen decrease while the contents in the float increase by increasing the time. This is not surprising, because a decrease of the calcium and sulfide contents in the hide have to be linked to an increase of calcium and sulfide in the float.

The initial Ca-content of the unhaired hide is 7.8 g/kg with a standard deviation of 0.69. The Ca-content of the conventional delimed hide is with 4.1 g/kg and a standard deviation of 0.05 about 50 wt% lower compared with the initial Ca-content.

After a process time of 25 min, the calcium content is with 5.25 g/kg about 20 wt% higher compared with the conventional delimed hide. However, by increasing the process time, the Ca-content in the hide decreases below the value of the conventional delimed hide already after 60 min. A further increase of the process time results in a decrease of the Ca-content. After 180 min, a nearly two times lower Ca-content compared with conventional delimed hide results. By decreasing the Ca-contents in the hide, the Ca-contents in the float increase from 0.4 to 0.8 g/kg by increasing the process time.

The reached Ca-contents confirm that a duration of 60 min for CO_2_-deliming at 30 bar including bating is sufficient to reach comparable results with a conventional deliming process of 2 h. It can be assumed that the residual Ca is associated to the anionic carboxy groups and would even remain inside the hide after longer deliming durations.

The initial sulfide content is 7.1 g/kg with a standard deviation of 0.4. The residual content of the conventional delimed hide is 6.1 g/kg with a standard deviation of 0.01. In the CO_2_-deliming process, the sulfide contents are between 4.5 and 5 g/kg independent from the process time. This indicates that an improved mass transport in the high-pressure process enables a fast sulfide removing. After 25 min, the influence on the decrease of sulfide is negligible. This is caused by the fact that some amino acids contain sulfur that is not washed out.

Depending on the pH-value, the remaining sulfides can form dangerous hydrogen sulfide. The concentration of hydrogen sulfide increases at pH-values below 9. To avoid the formation of hydrogen sulfide, oxidizing agents as hydrogen peroxide can be added (Deng et al. 2015). However, a tanning drum designed for high-pressure applications is sealed against the atmosphere. Therefore, all formed hydrogen sulfide remains in the process and does not get in contact with workers. Because the majority of the gas mixture within the process is non-flammable CO_2_, no explosive gas mixtures are formed. The portion of oxygen in the process chamber is negligible. During the depressurization, formed hydrogen sulfide can be separated from the gas stream without adding oxidizing agents that contaminate the wastewater. Possibly, the formed hydrogen sulfide can be used as feedstock for the production of sodium sulfide. Sodium sulfide is used for unhairing in the leather production chain (Quadery et al. 2014).

## Conclusion

We successfully performed salt-free CO_2_-deliming of unhaired hide in a pressurized autoclave at 30 bar. The tenseness of the CO_2_-delimed collagen fibers with pressurized CO_2_ is comparable with the tenseness of conventional delimed hide after a treatment of 60 min. A fast reduction of the tenseness is essential for an accelerated processing because the mass transport of water, lime, and sulfide increases by decreasing the tenseness. A residual Ca-content comparable with conventional delimed hide of about 0.4 g/kg based on the dry collagen weight was also reached within 1 h. The results show that a salt-free deliming by saving 50% of process time can be realized.

By the optical assessment of the pH-value in the view cell, we showed that an increased amount of water results in a decreased pH-value at the same pressure. This is explained by the higher availability of dissolved CO_2_ at a higher offer of water forming carbonic acid. However, because an unacceptable water consumption is needed to achieve a significant pH-decrease, we focused on a typical float length of 200 wt%.

Without buffering salts, the pH-value decreases from 12.5 to about 7 after reaching equilibrium at a float length of 200 wt% and pressures up to 30 bar. After 25 min, the difference in the pH-values between the float and the hide is 1. We assume that the pH-value after 25 min is stable enough to initiate bating. Enzymes that have a high activity between 6 and 8 to decompose non-collagen proteins are available. However, since we focused only on deliming, the influence of different bating enzymes on the leather properties operating at pH-values between 6 and 8 has to be assessed in additional work.

It has to be noted that in an industrial application, the formation of the dangerous hydrogen sulfide is promoted at the lower pH-value that results at 30 bar compared with conventional deliming. However, because the reaction happens in a sealed autoclave without the presence of oxygen, no additional chemicals as hydrogen peroxide have to be used. Hydrogen sulfide can be separated from the gas stream, transformed to sodium sulfide, and reused for unhairing.

In sum, the salt-free CO_2_-deliming enables an accelerated deliming of animal hides without any ammonium nitrogen emission. In combination with the CO_2_-intensified low chromium and sulfate emission tanning, CO_2_-deliming has high potential to enable environmental friendly industrial beamhouse and tanning operations.
